# The Effects of Varying Teacher-Student Ratios in a Special Education Classroom

**DOI:** 10.1007/s40617-025-01044-1

**Published:** 2025-01-31

**Authors:** Bianca N. Frost, Stephen F. Walker, Brandon C. Perez, Samantha A. Camacho, Natalie R. Andzik

**Affiliations:** 1 Turning Pointe Autism Foundation, Naperville, IL USA; 2 Behavioral Health and Counseling Department, St. Cloud State University, St. Cloud, MN USA; 3 Special and Early Education Department DeKalb, Northern Illinois University, DeKalb, IL USA

**Keywords:** Autism, Staff ratios, Special education, Momentary time sample, Classroom management

## Abstract

These procedures examined the impact of teacher–student ratios on staff and student behavior in a therapeutic day school. Ratios (teacher:student) of 5:4, 4:4, 3:4, and 2:4, involving the teacher, paraprofessionals, and four junior high students with autism were measured. Using momentary time sampling, environment, organization, student, and staff activity data were collected. Higher ratios correlated with increased student engagement and fewer behavioral interventions, while lower ratios led to decreased staff–student interactions and more behavioral interventions.

• Authors discussed the impacts of teacher–student ratios in educational settings for children with autism.

• State guidelines need revision to be sensitive to classroom composition.

• Researchers have focused on academic outcomes and teacher perceptions.

• Momentary time sampling is a practical approach for clinicians to use when objectively assessing variables in the classroom setting.

Researchers often manipulate and evaluate specific classroom variables to measure their impacts on student behavior and learning. After reviewing literature focused on classroom management, Kestner et al. (2019) identified four specific variables that have positive impacts on academic performance and student behavior; (a) pacing of activities, (b) appropriateness of the curriculum, (c) the use of feedback as reinforcement, and (d) the delivery of effective instructions and transitions. Although these variables are undoubtedly impactful for student outcomes, there is limited literature that investigates the effects of teacher–student ratios on staff and student behaviors. Furthermore, there is even less evidence of the effects of these ratios within special education settings, where teacher–student ratios are often critical to student success. Teacher retention is also often linked to workloads and working conditions, and decreased caseloads may make the role of a teacher more manageable (Peyton et al., 2021).

Each state’s legislators outline teacher–student ratio guidelines (e.g., State K-3 Policies 2023 Education Commission of the States*.* n.d.). For example, in Illinois, the teacher–student ratio within a general education classroom is independently determined by each school district. However, students who spend more than 60% of their day in a special education setting must be placed in a classroom that has at least an 1:8 teacher–student ratio, and the maximum number of students may increase depending on paraprofessional assistance (Illinois State Board of Education, ISBE, n.d.). In Ohio, ratios are determined by disability category and age (Ohio Department of Education, ODE, n.d.). For example, a special education teacher may serve no more than 16 children at the elementary and middle levels and no more than 24 students at the high school level. However, in Ohio, special education teacher’s ratio caps are lower by instructional period for children with emotional behavior disorders (10), hearing impairments, visual impairments, orthopedic impairments, multiple disabilities, and/or other health impairments (8), and autism, deaf–blindness and/or traumatic brain injury (6). Yssedlyke and colleagues (1989) were the last known research group to evaluate mean teacher–student ratios in special education classrooms nationwide. They reported that across 220 secondary-level special education classrooms in 35 states, schools had a mean teacher–student ratio of 1:5.3 (range 1:1.6–1:12).

Some researchers have focused their discussion and investigation around teacher–student ratios through teacher reports, surveys, and observations. For example, Johnston (1990) reported that general education teachers across grades K–3 reported a more manageable and rewarding teaching environment when compared to their colleagues who had larger class sizes. Although over 1000 teachers were surveyed, this author only measured perceived differences and did not document any student data. Thurlow et al. (1993) manipulated the ratios in special education classrooms and noted how the quality of instruction changed. Specifically, they measured (1) time on task, student teacher interactions, instructional activities, and classroom management. They found that reduced student teacher ratios resulted in more individualized instruction as well as teacher satisfaction. Other researchers did take into consideration some student level data when discussing the benefits to lower student teacher ratios. Odden (1990) reviewed the literature and found that smaller general education classrooms were related to higher student achievement and allowed for more differentiation for students who were at risk. Although Odden did suggest that teacher morale likely increased with smaller class sizes, the cost–benefit analysis was not as compelling as a system-wide class size reduction may not be cost effective in many places.

Even fewer researchers have reported student behavioral outcomes when evaluating teacher–student ratios. Field (1980) evaluated the effects of teacher–student ratios on classroom organization with 80 preschool students across four classrooms. A time sampling procedure was used to evaluate student behavior. Results indicated that given a high teacher–student ratio (i.e., 1:12) and small specific portioned play areas, children engaged in more optimal behaviors (e.g., interactions with peers, fantasy play). A potential limitation to this study was the researchers’ inability to control different teaching styles from one classroom to another. Also, owing to the specific demographic of participants, researchers reported that results would most likely not generalize to children with different backgrounds (e.g., children from lower socioeconomic statuses).

Russell (1990) evaluated the effects of teacher–student ratios on child and staff behavior in 27 preschools in Australia. A time sampling method was used as researchers scored the presence and absence of group organization, teacher–student interactions, and student play activities. Results indicated that although lower teacher–student ratios had little impact on staff behavior, they did decrease undesirable behaviors displayed by the children. Researchers did not collect interobserver agreement for behaviors that required “interpretation” or “judgment” by the observer. Schwartz et al. (2012) evaluated the effects of varying ratios on literacy skills with at-risk first graders. They found that a 1:1 instruction resulted in significantly higher literacy outcomes when compared to 1:2, 1:3, and 1:5. Although findings from these studies are meaningful, the data were captured decades ago, and few researchers have targeted how teacher–student ratios affect staff and student behavior within the classroom setting.

Some researchers have used momentary time sampling procedures to assess staff and client behavior and the quality of care in state residential facilities for individuals with developmental disabilities (Joslyn et al., 2017) and centers providing applied behavior analysis therapy to children with autism and other related disabilities (Grauerholz-Fisher et al., 2019). These researchers observed multiple aspects of the setting, such as environmental conditions, client conditions, client behavior, and staff behavior. The data collection procedure utilized in these studies was effective across multiple settings, activities, and behaviors. This suggests these procedures could be adjusted to assess staff and student behavior in school settings.

Given the lack of previous research evaluating how teacher–student ratios impact the behavior of students and staff, methods that allow for evaluating these varying ratios must be developed. These methods will allow individual schools and classrooms to create teacher–student policies tailored to individual classroom needs and eliminate the need for arbitrary blanket policies. The purpose of the current study was to evaluate the effects of teacher–student ratios on student and staff behavior by replicating and extending the work of Josyln et al. (2017) and Grauerholz-Fisher et al. (2019) by using a momentary time-sampling procedure to evaluate the effects of varying teacher–student ratios in a special education classroom within a therapeutic day school.

## Method

### Participants and Setting

Sessions were conducted within a junior high classroom at a therapeutic day school for children with a primary diagnosis of autism who had been referred to this school for the presence of severe problem behaviors that could not be managed within the public school setting. Four of the five students in the classroom were included in the study due to the specialized programming of one of the students. All students had a significant intellectual disability and an individualized education plan (IEP) stating that they benefit from a small class size (i.e., 6–7 students) and individual support. Bradley was a 14-year-old White boy who engaged in self-injury and physical aggression. Jason was a 13-year-old White boy who engaged in physical aggression, self-injury, and off-task behavior. Andrew was a 13-year-old White boy who engaged in physical aggression and tantrum behaviors. Tara was a 14-year-old Black girl who engaged in self-injury to the head, physical aggression, and emesis.

One licensed special education teacher and four paraprofessionals were included in the study. Staff members included four men and one woman and all had been employed at the day school for 7–29 months (*M* = 18.8). Staff experience prior to starting at this school varied from no experience to more than five years. The classroom teacher had a bachelor’s degree, one paraeducator had a master’s degree, two paraeducators had a bachelor’s degree and one had a high school diploma. All staff were trained using behavior skills training in behavior management systems and behavior support techniques which included training on student-specific programming, as well as staff expectations in the classroom and school as a whole.

All observations occurred during group reading lessons and consisted of presenting a nonfiction book (differentiated to their reading level, grade one) and follow-up comprehension questions led by the classroom teacher.

### Observation Procedures

Students and staff were observed up to four times daily. At the beginning of each day, the head teacher provided the daily schedule outlining the possible opportunities for observations to the first author. To control for extraneous variables, observation times and teacher–student ratios were selected using a randomized drawing. Once a ratio was chosen, it was removed from that day’s future drawings. Owing to state regulations requiring the presence of a staff member with a special education license, the head teacher participated in all observation sessions. Paraprofessionals participating in the observation were drawn before each session. Five minutes before the observation was to occur, the head teacher and staff were notified of the ratio and the staff participating in the observation.

The school’s board-certified Behavior Analyst (BCBA) and two graduate students trained in the data collection procedure conducted all observations. Observations only occurred on days when all participants were present. One observation of each learning session started ten minutes after a reading lesson had begun. A momentary time-sampling procedure was used to extend previous research and evaluate the effects of varying teacher–student ratios in a special education classroom within a therapeutic day school. The observer scored categories of classroom environment, classroom organization, student activity, and staff activity using a momentary time-sampling procedure. Observers did not interact with staff or students during data collection. Data collection sheets and definitions of targets were provided to observers and are available upon request from the corresponding author. Similar to Grauerholz-Fisher et al. (2019), there was not a specific time limit specified for each observation. When observations began, observers would enter a room and the primary data collector would indicate the student to be observed, scanning right to left. The order of variables observed for each student was: (a) classroom environment, (b) classroom organization, (c) student activity, and (d) staff activity. Once both observers scored each of the variables, the primary data collector would nod to the next student to be observed. This would continue until all students in the classroom had been observed and the observation session concluded.

#### Classroom Environment

The classroom environment was scored as the observer scanned the classroom from right to left specifically noting two areas, safety and supplies/materials. Observers first noted the total number of students in the room, followed by the total number of students who were displaying accurate responses. Safety was scored ( +) if the student was seated, has a clear pathway to classroom exit in case of emergency, and all sharp objects are out of reach, including scissors, pens, pencils if not in use. A score ( −) was given if a student was out of their seat, if the pathway to classroom exit was blocked, or if sharp objects were within reach, including scissors, pens, pencils, if not in use. Supplies/Materials score of ( +) was given if all materials were present (i.e. token economy, escalation visual, communication system, and student specific safety materials described in the student’s behavior plan). A ( −) score was given if any of the materials were missing.

#### Classroom Organization

All variables within classroom organization were scored as a permanent product. The classroom organization was scored as the observer scanned the classroom from right to left noting mini-schedules and completed expectations. Mini-schedules were scored ( +) if the student’s mini-schedule was present, listed session expectations, and was within the student’s visual field. A score of ( −) was noted if the mini-schedule was absent, did not list session expectations, or was outside of the student’s visual field. Among the completed expectations scores, a ( +) was noted if completed expectations were crossed off of the mini-schedule once completed by student. A ( −) score was noted if completed expectations had not been crossed off of the mini-schedule once completed by the student. Expectations represented on the mini-schedule were the number of pages being read aloud as a group and the number of comprehension questions the student was required to answer.

#### Student Activity

Student activity was scored as the observer scanned the classroom from right to left noting two areas, appropriate academic task and engaging in target problem behavior Appropriate academic tasks were scored ( +) if the student was engaging in a task/activity that was listed on the mini-schedule. For activities that did not require physical activity, such as reading, student eye gaze must have been on material(s). A score of ( −) was noted if the student was engaging in any task/activity that was not listed with an expectation displayed on the mini-schedule. For activities that did not require physical activity, such as reading, student eye gaze was not on the material(s). Engaging in problem behaviors included a ( +) score when the student was engaging in any target problem behavior listed in the student’s behavior intervention plan. These behaviors included: self-injury (Bradley: head to fixed object or knee to head; Jason: biting; Tara: closed or open fist to head), physical aggression (Bradley: head to person’s body; Jason: biting; Tara: scratching and/or pinching; Andrew: open hand to person’s body), off-task behavior (Jason: not engaging with or attending to assigned tasks), and tantrum behavior (Andrew: crying, whining, other loud vocalizations, flipping desk over, lying on the ground). A score of (–) was noted if the student was not engaging in any target problem behavior listed in the student’s behavior intervention plan.

#### Staff Activity

The classroom environment was scored as the observer scanned the classroom right to left noting three activities, including standing, appropriate student interaction, and behavioral intervention targets. Standing received a ( +) score if the staff was standing and a (–) if they were sitting. Appropriate student interaction included a ( +) score if the staff was interacting with at least one student in regard to an appropriate academic task. A (–) score was noted if the staff was not interacting with at least one student or interacting with students about something other than an appropriate academic task. Behavioral intervention scores included a ( +) if staff was engaging in any reactive behavior intervention protocol outlined in the student’s behavior intervention plan and a ( −) if the staff was not engaging in any reactive behavior intervention protocol outlined in the student’s behavior intervention plan.

### Classroom Ratios

The following teacher–student ratios were evaluated; 5:4, 4:4, 3:4, and 2:4. Ratio 5:4 included the classroom teacher, four paraprofessional staff members and four students. Ratio 4:4 included the classroom teacher, three paraprofessional staff members and four students. Ratio 3:4 included the classroom teacher, two paraprofessional staff members and four students. Ratio 2:4 included the classroom teacher, one paraprofessional staff member and four students. Each ratio was observed 10 times across the duration of the study.

### Data Calculation

For classroom environment, organization, student activity, staff activity, and all subcategories, percentage of observations was calculated by adding the total number of “ + ” responses observed, dividing by the total possible responses, and multiplying by 100. Percentage of observations was calculated for all observed teacher–student ratios.

### Reliability

Interobserver agreement was assessed by having a second observer conduct simultaneous but independent observations for 38% of sessions. During reliability observations, the lead observer would guide the second observer into the classroom and following the signal of a timer both observers would scan the room right to left. Each observer first observed the classroom environment, then classroom organization, followed by student activity, and finished the observation session with staff activity. Once the observers had completed assessing all categories and subcategories they would signal each other with a nod and simultaneously exit the classroom. Interobserver agreement was calculated for all categories by dividing the total number of agreements by the total number of agreements possible (both accuracy and violations). Mean agreement scores were 100%, for classroom environment, 100% for classroom organization, 98% for student activity (range 75–100%), and 97% (range 67–100%) for staff activity.

## Results

**Fig. 1:**
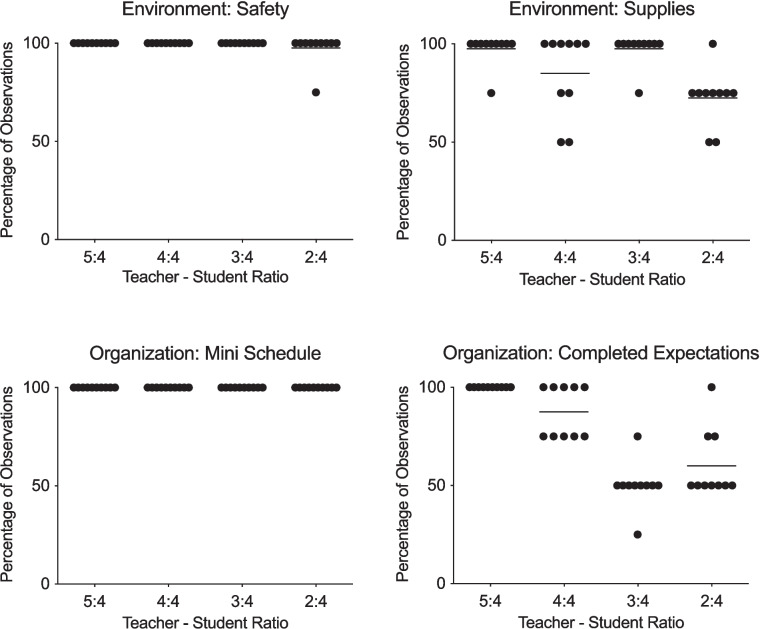
Results of teacher–student ratios on classroom environment and classroom organization

**Fig. 2:**
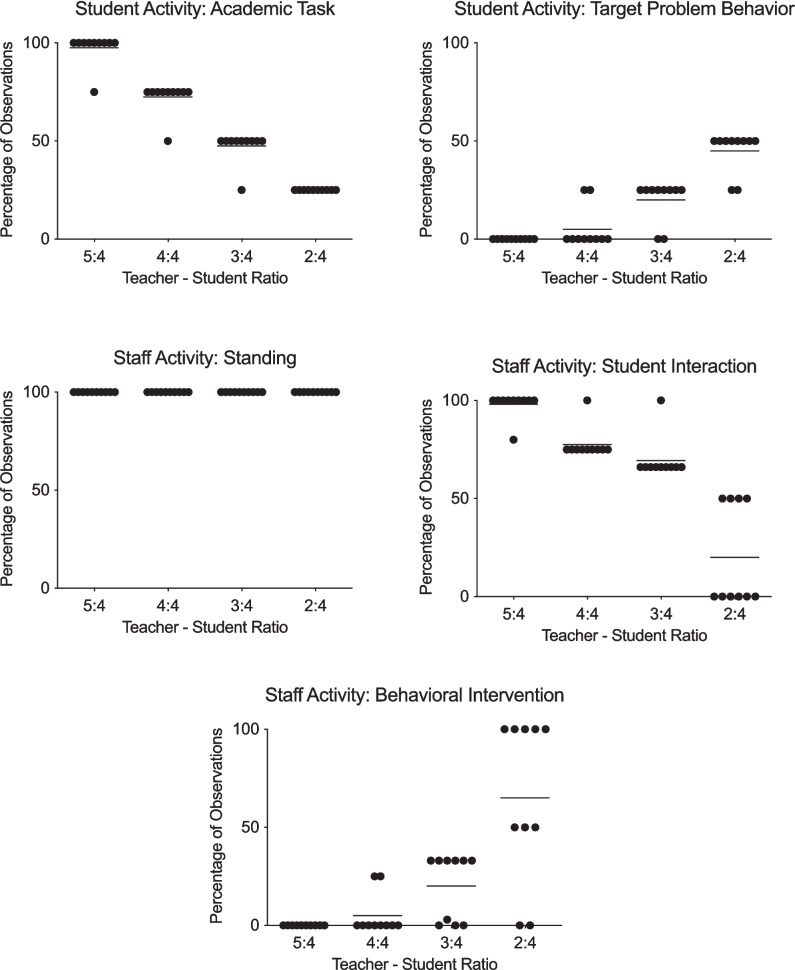
Results of teacher–student ratios on student activity and staff activity

Figure 1 displays the results of teacher–student ratios for classroom environment and organization. Figure 2 displays the results of teacher–student ratios for student activity and staff activity. The data points represent the percentage of observations for each session at the teacher–student ratio depicted on the x-axis. The horizontal lines represent the mean percentage of observations for each ratio across sessions. The top row of Fig. 1 depicts the environment conditions, safety and supplies. The bottom row depicts the organization conditions, mini-schedule and completed expectations. The top row of Fig. 2 depicts the student activity conditions, academic task and target problem behaviors. The middle row of graphs depict the staff activity conditions, standing and appropriate student interaction. The final row depicts the staff activity condition, behavioral intervention.

The mean percentage of observations for the safety measure was 100% at ratios 5:4, 4:4, 3:4, and 98% at ratio 2:4 (range 75–100%). The mean percentage of observations for the supplies/materials measure was 97.5% at ratio 5:4 (range 75–100%), 85% at ratio 4:4 (range 50–100%), 98% at ratio 3:4 (range 75–100%), and 73% at ratio 2:4 (range 50–100%).

The mean percentage of observations for the mini-schedule conditions was 100% at ratios 5:4, 4:4, 3:4, and 2:4. The mean percentage of observations for the completed expectations measure was 100% at ratio 5:4, 88% at ratio 4:4 (range 75–100%), 50% at ratio 3:4 (range 25–100%), and 60% at ratio 2:4 (range 50–100%). The mean percentage of observations for the appropriate academic task measure was 98% at ratio 5:4 (range 75–100%), 73% at ratio 4:4 (range 50–75%), 48% at ratio 3:4 (range 25–50%), and 25% at ratio 2:4. The mean percentage of observations for problem behavior was 0% at ratio 5:4, 5% at ratio 4:4 (range 0–25%), 20% at ratio 3:4 (range 0–25%), and 45% at ratio 2:4 (range 25–50%).

The mean percentage of observations for the standing condition was 100% at ratios 5:4, 4:4, 3:4, and 2:4. The mean percentage of observations for appropriate student interaction was 98% at ratio 5:4 (range 80–100%), 78% at ratio 4:4 (range 75–100%), 69% at ratio 3:4 (range 66–100%), and 20% at ratio 2:4 (range 0–50%). The mean percentage of observations for behavioral intervention was 0% at ratio 5:4, 5% at ratio 4:4 (range: 0–25%), 23% at ratio 3:4 (range 0–33%), and 65% at ratio 2:4 (range 0–100%).

## Discussion

The purpose of the current study was to empirically evaluate the effects of varied teacher–student ratios on staff and student behavior in a special education classroom. The results of this study show that students engaged in fewer academic tasks and more problem behavior with lower teacher–student ratios, and staff engaged in more reactive behavioral interventions and less appropriate student interactions with lower teacher–student ratios. These results are reasonable as staff could not engage in appropriate student interactions while students engaged in problem behavior, and reactive behavioral interventions were needed.

By replicating and expanding on the use of momentary time sampling as an objective measurement procedure, as done by Josyln et al. (2017) and Grauerholz-Fisher et al. (2019), these data represent an unbiased picture of the impacts of varying teacher–student ratios on staff and student behavior. This addresses the gap in previous research, which relied heavily on anecdotal accounts, such as those seen by Russell (1990), who reported that observers failed to agree on behavior occurrences when definitions called for personal “judgment” or “interpretation.” The observable definitions utilized by the authors resulted in high interobserver agreement across all observation definitions with the expectations of staff activity which may be due to the increase of uncontrolled variables among staff, leaving room for interpretation. In addition, the current study achieved high levels of participant and environmental control resulting in reliable data collection. Student participation remained constant throughout the study while a limited pool of five staff was used to manipulate ratios. This level of control addresses the limitation of Field (1980), stating researchers could not control for differing teaching styles when evaluating teacher–student ratios and classroom structure.

A few limitations of the current study should be considered. First, there was no control over which staff members were included in each ratio outside of the presence of the head teacher (i.e., selection of staff that left the classroom was random). Varying training, years of experience, and education among staff members could affect accuracy of responding. The authors did observe that specific staff were more (or less) likely to engage in appropriate responses during observations, which may have implications for ongoing monitoring and staff training. Future research should evaluate the effects of consistent dyads of staff and students when evaluating the effects of teacher–student ratios on staff and student behavior. Second, observations only occurred during one activity (i.e., reading sessions) and at author-driven predetermined times. It is possible that the current results would not be replicated during different or less structured activities. Additionally, it may not be feasible to manipulate a classroom’s daily schedule for observations to occur at specified times within a less restrictive educational setting. Future research should evaluate the effects of teacher–student ratios on staff and student behavior across a variety of education activities and settings (e.g., lunchtime, physical education).

In closing, it is often unclear how school districts and statewide stakeholders develop policies around teacher–student ratios in special education settings. The goal of the current study was not only to provide a general account of how teacher–student ratios impact staff and student behavior within a therapeutic day school, but more importantly evaluated a methodology for administrators to assess and establish appropriate teacher–student ratios within specific classroom settings. The current study is not without its limitations. For example, it is likely that the dense ratios that were beneficial for this population, due to their high level of need, may not yield the same results for students in less restrictive settings where the emphasis on education is age-appropriate curriculum and building academic independence. We encourage the customization and replication of the methods of this study as they may be a starting point for data-driven decisions regarding teacher–student ratios within special education classrooms moving forward.

## References

[CR1] Field, T. M. (1980). Preschool Play: Effects of Teacher/Child Ratios and Organization of Classroom Space. *Child Study Journal,**10*(3), 191–205.

[CR2] Grauerholz-Fisher, E., Vollmer, T. R., Peters, K. P., Perez, B. C., & Berard, A. M. (2019). Direct assessment of quality of care in an Applied Behavior Analysis Center. *Behavioral Interventions,**34*(4), 451–465. 10.1002/bin.1680

[CR3] Illinois State Board of Education (ISBE). (n.d.). https://www.isbe.net/Documents/226ark.pdf. Accessed 18 Dec 2024.

[CR4] Johnston, J. M. (1990). *Relations between reduced class size and reduced teacher/pupil ratio and developmentally appropriate practice in kindergarten through third grades.* Paper presented at the Annual Meeting of the American Educational Research Association, Boston, MA.

[CR5] Joslyn, P. R., Vollmer, T. R., Dickens, E. N., & Walker, S. F. (2017). Direct assessment of quality of care in secure residential treatment facilities for criminal offenders with intellectual disabilities. *Behavioral Interventions,**33*(1), 13–25. 10.1002/bin.1501

[CR6] Kestner, K. M., Peterson, S. M., Eldridge, R. R., & Peterson, L. D. (2019). Considerations of Baseline Classroom Conditions in Conducting Functional Behavior Assessments in School Settings. *Behavior Analysis in Practice,**12*(2), 452–465. 10.1007/s40617-018-0269-1 31976253 10.1007/s40617-018-0269-1 PMC6745578

[CR7] Odden, A. (1990). Class size and student achievement: Research-based policy alternatives. *Educational Evaluation and Policy Analysis,**12*(2), 213–227. 10.3102/01623737012002213

[CR8] Ohio Board of Education (ODE, n.d.). https://codes.ohio.gov/ohio-administrative-code/rule-3301-51-09#:~:text=(b)%20An%20intervention%20specialist%20shall,during%20any%20one%20instructional%20period.

[CR9] Peyton, D. J., Acosta, K., Harvey, A., Pua, D. J., Sindelar, P. T., Mason-Williams, L., Dewey, J., Fisher, T. J., & Crews, E. (2021). Special education teacher shortage: Differences between high and low shortage states. *Teacher Education and Special Education,**44*(1), 5–23. 10.1177/0888406420906618

[CR10] Russell, A. (1990). The effects of child-staff ratio on staff and child behavior in preschools: An experimental study. *Journal of Research in Childhood Education,**4*(2), 77–90.

[CR11] Schwartz, R. M., Schmitt, M. C., & Lose, M. K. (2012). Effects of teacher-student ratio in response to intervention approaches. *The Elementary School Journal,**112*(4), 547–567.

[CR12] State K-3 Policies 2023 - Education Commission of the States*.* (n.d.). Reports.ecs.org. https://reports.ecs.org/comparisons/state-k-3-policies-2023-09

[CR13] Thurlow, M. L., Ysseldyke, J. E., Wotruba, J. W., & Algozzine, B. (1993). Instruction in special education classrooms under varying student-teacher ratios. *The Elementary School Journal,**93*(3), 305–320. 10.1086/461736

[CR14] Ysseldyke, J. E., Thurlow, M. L., & Wotruba, J. W. (1989). Special education student-teacher ratios for mildly handicapped children. *The Journal of Special Education,**23*(1), 95–106.

